# Prediction of major adverse cardiovascular events with two risk scales for acute chest pain in the emergency department

**DOI:** 10.47487/apcyccv.v6i4.555

**Published:** 2025-12-29

**Authors:** Jocabed Miranda-Chávez, José Amado-Tineo

**Affiliations:** 1 Hospital Nacional Edgardo Rebagliati Martins, EsSalud, Lima, Perú. Hospital Nacional Edgardo Rebagliati Martins EsSalud Lima Perú

**Keywords:** Chest Pain, Cardiovascular Diseases: Forecasting, Diagnosis, Dolor Torácico, Enfermedades Cardiovasculares, Predicción, Diagnóstico

## Abstract

**Objectives.:**

To compare the ability of the HEART and EDACS scores to predict major adverse cardiovascular events (MACE) at 30 days of follow-up in patients with acute chest pain presenting to an emergency department.

**Materials and Methods.:**

Retrospective study of patients older than 18 years treated for acute chest pain, excluding ST-elevation acute coronary syndrome (ACS), trauma, and infections. The HEART and EDACS scores were assessed at admission. The area under the receiver operating characteristic curve (AUC), sensitivity, specificity, positive predictive value, and negative predictive value of both scores were calculated for the prediction of 30-day MACE.

**Results.:**

A total of 249 patients were evaluated; 62.2% were male, with a mean age of 66.5 years. There were 25 MACEs (10%). The HEART score classified patients as low risk (43.4%), moderate risk (47.4%), and high risk (9.2%). Using the EDACS, patients were classified as low risk (38.6%) and not low risk (61.4%). Regarding MACE, the HEART score had an AUC of 0.91 (95% CI: 0.87-0.95) and EDACS had an AUC of 0.70 (95% CI: 0.60-0.79). The HEART score demonstrated better performance than EDACS, especially when a score ≥4 was obtained.

**Conclusions.:**

The HEART score has higher diagnostic performance than EDACS for predicting MACE in patients with acute chest pain presenting to a tertiary emergency department.

## RESUMEN

**Objetivos.:**

Comparar la capacidad de las escalas HEART y EDACS para la predicción de eventos adversos cardiovasculares mayores (MACE) a los 30 días de seguimiento en pacientes con dolor torácico agudo que acuden a un servicio de emergencia.

**Materiales y métodos.:**

Estudio retrospectivo en mayores de 18 años atendidos por dolor torácico agudo, excluyendo síndrome coronario agudo (SCA) con elevación del ST, traumatismos e infecciones. Se evaluaron las escalas HEART y EDACS al ingreso. Se calculó el área bajo la curva (ABC) de características operativas del receptor (ROC), sensibilidad, especificidad, valor predictivo positivo y valor predictivo negativo de ambas escalas en la predicción de MACE a 30 días.

**Resultados.:**

Se evaluó 249 pacientes, 62,2% varones, edad promedio 66,5 años. Se encontraron 25 MACEs (10%). La escala HEART clasificó a los pacientes en riesgo bajo (43,4%), moderado (47,4%) y alto (9,2%). Con la escala EDACS se encontró pacientes de bajo riesgo (38,6%) y no bajo riesgo (61,4%). En relación a los MACE, la escala HEART tuvo un ABC de 0,91 (IC 95%: 0,87 - 0,95) y el EDACS de 0,7 (IC 95%: 0,60 - 0,79). La escala HEART obtuvo mejor rendimiento que EDACS, especialmente cuando se obtuvo un puntaje >=4.

**Conclusiones.:**

La escala HEART tiene mayor rendimiento diagnóstico que EDACS para predecir MACE en pacientes con dolor torácico agudo que acuden a un servicio de emergencia.

## Introduction

Acute chest pain is a common presenting symptom in the emergency department. Among its aetiologies, angina-type chest pain raises concern for a possible acute coronary syndrome (ACS), which must be managed promptly according to the degree of haemodynamic compromise it produces in the patient ^1^^-^^3^.

The wide range of causes of acute chest pain compels emergency physicians to optimise resources in order to determine both the risk of ACS and its severity ^4^. Tzu-Yun reported the unnecessary use of human and material resources in cases of acute chest pain driven solely by the “fear of missing an acute myocardial infarction”, leading to inappropriate testing, prolonged waiting times, unnecessary consultations, and extended observation periods; improvements were observed with the implementation of acute chest pain risk scores ^5^.

The HEART score (History, Electrocardiogram, Age, Risk factors, and Troponin) incorporates variables that are readily obtainable, including the clinical characteristics of typical or atypical chest pain, electrocardiographic findings, age, cardiovascular risk factors, and troponin levels. This score was developed in the Netherlands in 2008 to differentiate patients with acute chest pain who have non-ST-segment elevation myocardial infarction (excluding those with ST-segment elevation myocardial infarction, given the overt electrocardiographic features) from those with non-coronary diagnoses ^6^^,^^7^.

Prospective validation of the HEART score demonstrated that discharging low-risk patients (HEART score <4) is safe, with only 0.6% experiencing major adverse cardiovascular events within 30 days of follow-up ^6^. When compared with the TIMI (Thrombolysis in Myocardial Infarction) score and a modified HEART score (excluding patient age), no statistically significant differences were observed in logistic regression analyses. Although the modified HEART score and the HEART pathway showed better performance, both require repeat measurements of high-sensitivity troponin, making them less practical in routine clinical settings ^7^.

The EDACS (Emergency Department Assessment of Chest Pain Score) also includes easily accessible variables such as age, sex, prior coronary artery disease, cardiovascular risk factors, and pain characteristics. However, it does not incorporate electrocardiographic findings and relies primarily on clinical history. It was developed in the emergency department to discriminate patients at risk of major adverse cardiovascular events within 30 days ^8^. Boyle *et al.* conducted a systematic review evaluating the sensitivity of EDACS in identifying patients with angina-type acute chest pain associated with major adverse events in the emergency department, reporting safe early discharge in up to 50% of cases. Although a second troponin measurement at two hours was included, their findings support the use of this score ^8^.

The most commonly used emergency department chest pain risk scores (HEART, HEART pathway, EDACS, ADAPT, mADAPT, NOTR, Vancouver, among others) structure the patient evaluation process by incorporating clinical history and objective data to ensure a low risk of myocardial infarction or major adverse cardiovascular events at discharge, thereby avoiding unnecessary investigations and reducing hospital admissions ^1^^,^^9^. However, controversy persists regarding which score offers the greatest sensitivity and specificity. Therefore, the present study aims to compare the performance of two of these scores (HEART and EDACS) in coronary risk stratification among adult patients presenting with acute chest pain in a tertiary referral emergency department.

## Materials and methods

### Study design and population

This was a retrospective observational study conducted among patients presenting with acute chest pain to the adult emergency department of Hospital Nacional Edgardo Rebagliati Martins (Lima, Peru), a social security-affiliated tertiary hospital providing approximately 200,000 emergency visits per year. Patients with a diagnosis of chest pain (ICD-10 R07.X) recorded in the institutional electronic medical record during 2022, who had undergone high-sensitivity troponin testing and were aged 18 years or older, were included. Patients with ST-segment elevation acute coronary syndrome on electrocardiography, a history of trauma, fever, encephalopathy, or incomplete data were excluded.

### Study variables

For risk stratification, the following variables were identified: sex, age, comorbidities, cardiovascular risk factors, symptoms, duration of symptoms, and high-sensitivity cardiac troponin T levels (Elecsys TnT-hs STAT cobas®; normal value: <14 ng/L). HEART score cut-offs were defined as low risk (0-3 points), intermediate risk (4-6 points), and high risk (7-10 points); EDACS categories were defined as low risk (<16 points) and non-low risk (≥16 points).

Major adverse cardiovascular events (MACE) were defined as myocardial infarction, emergency surgical or percutaneous coronary revascularisation, and death. The association between the risk scores and the development of ACS was also assessed.

### Procedures

Due to a high proportion of incomplete records, a random sample of 500 patients was drawn from the list of individuals who attended with an R07.X diagnosis in 2022. Two specialist physicians independently reviewed the electronic medical records, calculated HEART and EDACS scores at emergency department admission, and were blinded to final outcomes. Admission diagnoses and the occurrence of MACE within 30 days were subsequently recorded.

### Ethical aspects

The study was approved by the institutional ethics committee (approval letter No. 384-GRPR-ESSALUD-2023). The principles of the Declaration of Helsinki and good research practice guidelines were followed, ensuring patient confidentiality. Informed consent was not required because data were obtained from electronic records, with no direct interaction with patients or their relatives.

### Data analysis

Sample size was calculated using the formula for comparison of proportions, yielding a minimum of 235 participants. Parameters were estimated assuming a 12.8% prevalence of MACE, a HEART score sensitivity of 97.4%, specificity of 54.2% (7), a 5% margin of error, and a 95% confidence level, using Epi Info version 7.2.5.

Categorical variables were summarised as frequencies and percentages, and numerical variables as measures of central tendency and dispersion according to their distribution. In bivariate analyses, chi-square or Fisher’s exact tests were used to assess differences between proportions, with p<0.05 considered statistically significant. Receiver operating characteristic (ROC) curves and areas under the curve (AUC) with 95% confidence intervals (CI) were calculated using SPSS version 27. Sensitivity, specificity, positive predictive value, and negative predictive value of the HEART and EDACS scores for identifying MACE and ACS at 30 days were also estimated, with corresponding 95% CI.

## Results

A total of 3,186 emergency department visits in 2022 met the proposed diagnostic code and age criteria. Upon review of the electronic medical records of the 500 patients selected by simple random sampling, 146 did not correspond to the recorded diagnosis, 56 had no electrocardiogram documented, 32 had no troponin measurement, 9 were duplicate cases, and 8 had ST-segment elevation myocardial infarction.

Overall, 249 patients met the inclusion criteria; 62.2% were male, and age ranged from 18 to 97 years (mean: 66.5 ± 14.5 years). The most frequent cardiovascular risk factors were hypertension, previous coronary artery disease, and diabetes mellitus. The most common comorbidities, in addition to previous coronary artery disease, were heart failure and cancer (Table 1).

**Table 1:** Characteristics of patients presenting with acute chest pain to the adult emergency department of a tertiary referral hospital

Characteristic	N=249 (%)
Age, years, mean (SD)	66.5 (14.5)
Male sex	155 (62.2)
Risk factors	2 (5,3%)
Previous coronary artery disease	48 (19.3)
Diabetes mellitus	67 (26.9)
Hypertension	142 (57.0)
Smoking	9 (3.6)
Obesity	6 (2.4)
Comorbidities	
Arrhythmias	22 (8.8)
COCD	85 (34.1)
Heart failure	23 (9.2)
Cancer	24 (9.6)
Chronic kidney disease	16 (6.4)
Hypothyroidism	15 (6.0)
Cerebrovascular disease	8 (3.2)
Rheumatological diseases	7 (2.8)
Chronic pulmonary disease	7 (2.8)
Mode of onset	
Sudden	59 (23.6)
Insidious	76 (30.5)
Not specified	95 (38.2)

SD: standard deviation. COCD: Chronic obstructive coronary disease.

The median duration of symptoms was 15 hours Chest pain was described as oppressive in 161 patients (64.7%), stabbing in 51 cases (20.5%), of another type in 10 cases (4.0%), and not recorded in 28 cases (10.8%). Pain onset was sudden in 23.6%, insidious in 30.5%, and not documented in 46.0%. Pain course was reported as continuous in 26.1%, progressive in 20.5%, intermittent in 15.3%, and not recorded in 38.1%. A history of prior angina was present in 38.8% of patients, 20.4% were receiving nitrates, and 175 patients (70.3%) had electrocardiographic abnormalities. Troponin levels were elevated in 79 cases (31.7%) and increased on repeat testing in 4.4%, with follow-up measurements performed in 40 patients (16.1%).

The HEART score ranged from 0 to 9, classifying 43.4% of patients as low risk, 47.4% as intermediate risk, and 9.2% as high risk (Table 2). EDACS scores ranged from -4 to 38, categorising 38.6% of participants as low risk and 61.4% as non-low risk (Table 3).

**Table 2:** Distribution of risk categories according to the HEART score in patients presenting with acute chest pain to the adult emergency department of a tertiary referral hospital.

category	Score	N (%)
HEART		
-H: history		
Typical chest pain	(2)	32 (12.9)
Features of both typical and atypical chest pain	(1)	76 (30.5)
Atypical chest pain only	(0)	141 (56.6)
- E: electrocardiogram		
ST-segment depression	(2)	16 (6.4)
Non-specific repolarisation abnormalities	(1)	76 (30.5)
Normal	(0)	157 (63.1)
- A: age		
>65 years	(2)	155 (62.2)
45-65 years	(1)	74 (29.7)
<45 years	(0)	20 (8.0)
- R: risk factors		
3 atherosclerotic risk factors	(2)	45 (18.1)
1 or 2 risk factors	(1)	138 (55.4)
No risk factors	(0)	66 (26.5)
- T: troponin		
>3 times the upper limit of normal	(2)	30 (12.0)
1-3 times the upper limit of normal	(1)	49 (19.7)
<1 time the upper limit of normal	(0)	170 (68.3)
HEART score		
Low risk	0 - 3 puntos	108 (43.4)
Intermediate risk	4 - 6 puntos	118 (47.4)
High risk	7 - 10 puntos	23 (9.2)

HEART: History, Electrocardiogram, Age, Risk factors, Troponin.

**Table 3:** Distribution of risk categories according to the EDACS score in patients presenting with acute chest pain to the adult emergency department of a tertiary referral hospital.

Score categories	Criteria	N (%)
EDACS		
- Age (years):		
18-45	(+2)	20 (8.0)
46-50	(+4)	5 (2.0)
51-55	(+6)	17 (6.8)
56-60	(+8)	26 (10.4)
61-65	(+10)	34 (13.7)
66-70	(+12)	41 (16.5)
71-75	(+14)	36 (14.5)
76-80	(+16)	30 (12.0)
81-85	(+18)	21 (8.4)
>86	(+20)	16 (6.4)
- Sex. Male	(+6)	155 (62.2)
- Known coronary artery disease*	(+4)	117 (47.0)
- Chest pain characteristics		
Diaphoresis	(+3)	23 (9.2)
Radiation to arm, shoulder, neck or jaw	(+5)	76 (30.5)
Onset or worsening with inspiration	(-4)	62 (24.9)
Reproducible on palpation	(-6)	41 (16.5)
EDACS	<16 puntos	96 (38,6)
Low risk	<16 points	96 (38.6)
Non-low risk	≥16 points	153 (61.4)

* Previous myocardial infarction, coronary artery bypass grafting, or percutaneous coronary intervention and/or risk factors (dyslipidaemia, diabetes, hypertension, current smoking, family history of premature coronary artery disease, only in patients aged 18-50 years).

EDACS: Emergency Department Assessment of Chest Pain Score.

All patients were assessed by an emergency medicine specialist, and 54.2% (135 patients) also underwent cardiology evaluation. ACS was diagnosed in 49 cases (19.7% of the total), including 25 non-ST-segment elevation myocardial infarctions and 24 cases of unstable angina. MACE were identified in 25 patients (10%), all of which were myocardial infarctions, including one case of sudden cardiac death.

**Figure 1:**
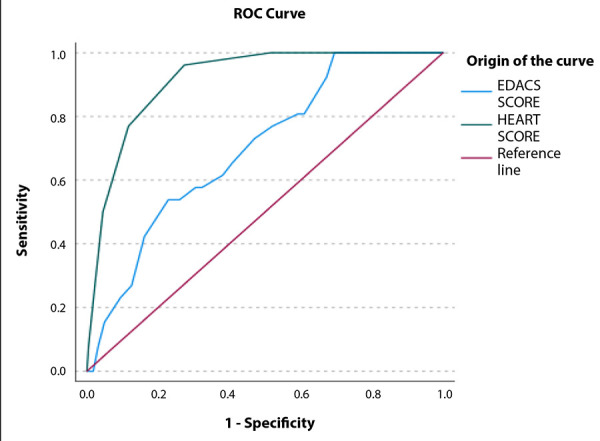
Area bajo la curva de las escalas HEART y EDACS para predecir eventos adversos cardiovasculares mayores (MACE) en pacientes atendidos por dolor toracico agudo en emergencia de adultos de un hospital referencial.

In the assessment of diagnostic performance for predicting MACE, the HEART score showed an AUC of 0.915 (95% CI: 0.87-0.95), whereas EDACS yielded an AUC of 0.70 (95% CI: 0.60-0.79; p<0.01) (Figure 1).

EDACS: Emergency Department Assessment of Chest Pain Score. HEART: History, Electrocardiogram, Age, Risk factors, Troponin.

For MACE prediction, the HEART score demonstrated maximal sensitivity (100%) when using the non-low-risk threshold (≥4 points) and high specificity (96%) when applying the high-risk threshold (≥7 points). EDACS showed a sensitivity of 81% for MACE, with a specificity of 41% (Table 4 and 5).

**Table 4:** Performance characteristics of risk stratification scores for major adverse cardiovascular events (MACE) in patients presenting with acute chest pain to the adult emergency department of a tertiary referral hospital.

Category	MACE at 30 days		Total
	Yes	No	n
HEART			
Low risk	0 (0)	108 (100.0)	108
Intermediate risk	13 (11.0)	105 (89.0)	118
High risk	13 (56.5)	10 (43.5)	23
EDACS			
Low risk	5 (5.2)	91 (94.8)	96
Non-low risk	21 (13.7)	132 (86.3)	153

MACE: major adverse cardiovascular events. HEART: History, Electrocardiogram, Age, Risk factors, Troponin. EDACS: Emergency Department Assessment of Chest Pain Score.

**Table 5:** Accuracy indicators of risk stratification scores for major adverse cardiovascular events (MACE) in patients presenting with acute chest pain to the adult emergency department of a tertiary referral hospital.

Score	Sensitivity (95%CI)	Specificity (95% CI)	PPV	NPV	LR+	LR-
HEART non-low risk (≥4 points)	100 (87.8-100)	48.4 (41.7-55.2)	18.4	100	1.92	0
HEART high risk (≥7 points)	50 (32.1-67.9)	95.5 (91.9-97.5)	56.5	94.2	0.57	0.52
EDACS non-low risk (≥16 points)	80.8 (62.1-91.5)	40.8 (34.6-47.4)	13.7	94.8	1.37	0.46

MACE: major adverse cardiovascular events. HEART: History, Electrocardiogram, Age, Risk factors, Troponin. EDACS: Emergency Department Assessment of Chest Pain Score. PPV: positive predictive value. NPV: negative predictive value. LR+: positive likelihood ratio. LR−: negative likelihood ratio.

## Discussion

Chest pain is a challenging symptom in the emergency department. The present study was conducted in a tertiary referral hospital with a substantial caseload. When risk stratification scores were calculated at presentation, both HEART and EDACS identified moderate to high coronary risk, with MACE confirmed in 10% of all patients. MACE were more frequent among men, older adults, and individuals with a history of hypertension, coronary artery disease, and diabetes mellitus, consistent with international reports. These findings provide locally generated evidence on the performance of the prognostic scores evaluated.

Chest pain is the cardinal symptom of cardiovascular disease and, at the same time, one of the most common complaints in emergency departments ^4^, encompassing not only cardiovascular emergencies but also musculoskeletal, respiratory, and other urgent conditions. Therefore, accurate characterisation of chest pain is essential to establish diagnostic suspicion and, in many cases, to exclude potentially life-threatening conditions such as ACS, which accounts for 5.1% of emergency department visits for chest pain in the United States and is responsible for more than 365,000 deaths annually ^4^. In Peru, national registries indicate the need to improve care pathways for patients diagnosed with myocardial infarction ^10^, with coronary heart disease accounting for 37% of deaths between 2017 and 2022, as reported by Quezada *et al.* in the Peruvian cardiovascular mortality registry ^11^.

Several risk scores have been validated for stratifying risk in patients with ACS (TIMI, GRACE, etc.) and acute chest pain (HEART, EDACS, etc.) ^12^^,^^13^. These tools support clinical decision-making, facilitate safe discharge, guide further invasive testing, or prompt early scheduling of therapeutic procedures ^1^^,^^4^. HEART and EDACS were selected for evaluation because they incorporate clinical variables, require few additional tests, are readily applicable, and have demonstrated discriminatory capacity in emergency settings ^13^^-^^15^.

In Peru, high sensitivity and specificity of the modified HEART score for predicting MACE have been reported in a private healthcare setting with a smaller population than that included in the present study ^16^. Although not explicitly mentioned in recent chest pain management guidelines, the HEART score demonstrates superior diagnostic performance, integrating clinical, electrocardiographic, and troponin variables that improve prediction compared with clinical assessment alone ^17^^,^^18^.

The EDACS score was designed to assess coronary risk using only clinical characteristics and cardiovascular risk factors in the emergency department, without incorporating laboratory data or other risk scores. Although high sensitivity has been reported, specificity is lower ^19^. This feature may be advantageous in settings without access to troponin testing. However, in institutions where this diagnostic tool is available, application of the HEART score appears more appropriate.

Regarding diagnostic performance, previous studies have shown that the HEART score has a higher AUC than TIMI and GRACE ^13^^,^^18^. In the present study, AUC values similarly indicate that HEART outperforms EDACS for predicting MACE, consistent with findings reported by Stopyra *et al.* in 2020 ^20^. Using the non-low-risk threshold (≥4 points), the HEART score achieved a sensitivity of 100% for MACE, comparable to the 95.9% reported in a meta-analysis including 44,202 patients from 30 studies published up to 2018, with similar specificity (48% in the present study vs. 44.6% in the meta-analysis) ^17^^,^^21^. This threshold allows identification of most at-risk patients, minimising unsafe discharge while optimising resource use by avoiding unnecessary hospitalisation of low-risk individuals. Nevertheless, these findings are influenced by patient case-mix, as referral centres receive more complex patients, limiting generalisability.

At the high-risk HEART threshold (≥7 points), specificity increases substantially but sensitivity declines markedly, consistent with previous reports ^16^^,^^21^. Considering likelihood ratios, a positive likelihood ratio greater than 1 and the lowest negative likelihood ratio correspond to the non-low-risk threshold (≥4 points), indicating superior overall test validity.

The sensitivity observed for EDACS in this study (81%) was lower than previously reported values of 96-99% ^8^^,^^19^. Similarly, specificity was lower (41%) compared with 50-61% reported in a 2020 meta-analysis including 11,578 patients ^8^. These differences are likely attributable to the higher clinical complexity of patients treated in a referral institution. Nonetheless, EDACS demonstrated acceptable validity for predicting MACE, particularly given that it does not incorporate troponin measurements and relies solely on clinical criteria and risk factors, consistent with its intended use in resource-limited settings.

The main limitations of this study include its retrospective design, a high proportion of missing or incomplete data, inability to determine referral status from other healthcare facilities, imprecise symptom onset times, and limited repeat troponin measurements. Despite being conducted at a single centre, the study included a substantial sample size. The hospital receives a high proportion of referrals from primary and secondary care facilities, typically involving complex cases; therefore, results may not be generalisable to lower-level healthcare settings. Importantly, this study represents one of the few publications providing local evidence on this topic.

Validation of these and other prognostic scores within each clinical context is essential to develop evidence-based protocols and clinical practice guidelines that improve diagnostic timeliness, enable prompt treatment, and optimise available resources, as demonstrated in other centres, where hospitalisation rates were reduced from 43% to 21.3% without an increase in 30-day MACE ^20^.

In conclusion, the HEART risk stratification score demonstrates superior diagnostic performance compared with EDACS for predicting MACE at 30 days among adult patients presenting with acute chest pain to a tertiary emergency department.

## References

[1] Market D, Marill KA, Schmidt A (2017). Identifying Emergency Department Patients With Chest Pain Who Are at Low Risk for Acute Coronary Syndrome. Emerg Med Pract.

[2] Byrne RA, Rossello X, Coughlan JJ, Barbato E, Berry C, Chieffo A (2023). 2023 ESC Guidelines for the management of acute coronary syndromes Developed by the task force on the management of acute coronary syndromes of the European Society of Cardiology (ESC). European Heart Journal.

[3] Instituto de Evaluación de Tecnologías en Salud e Investigación (IETSI) (2017). Guía de Práctica Clínica de Síndrome Isquémico Coronario Agudo. Guía en Versión Corta. GPC N°4.

[4] Gulati M, Levy PD, Mukherjee D, Amsterdam E, Bhatt DL, Birtcher KK (2021). 2021 AHA/ACC/ASE/CHEST/SAEM/SCCT/SCMR Guideline for the Evaluation and Diagnosis of Chest Pain A Report of the American College of Cardiology/American Heart Association Joint Committee on Clinical Practice Guidelines. Circulation.

[5] Liu TY, Tsai MT, Chen FC, Pan HY, Huang JB, Cheng FJ (2021). Impact of coronary risk scores on disposition decision in emergency patients with chest pain. Am J Emerg Med.

[6] Halder D, Mathew R, Jamshed N, Yadav S, Rl B, Aggarwal P (2021). Utility of HEART Pathway in Identifying Low-Risk Chest Pain in Emergency Department. J Emerg Med.

[7] Kim MJ, Ha SO, Park YS, Yi JH, Yang WS, Kim JH (2021). Validation and modification of HEART score components for patients with chest pain in the emergency department. Clin Exp Emerg Med.

[8] Boyle RSJ, Body R (2021). The Diagnostic Accuracy of the Emergency Department Assessment of Chest Pain (EDACS) Score A Systematic Review and Meta-analysis. Ann Emerg Med.

[9] Reynolds HR, Shaw LJ, Min JK, Spertus JA, Chaitman BR, Berman DS (2020). Association of Sex With Severity of Coronary Artery Disease, Ischemia, and Symptom Burden in Patients With Moderate or Severe Ischemia Secondary Analysis of the ISCHEMIA Randomized Clinical Trial. JAMA Cardiol.

[10] Ríos P, Pariona M, Urquiaga JA, Mendez FJ (2020). Características clínicas y epidemiológicas del infarto de miocardio agudo en un hospital peruano de referencia. Rev Perú Med Exp Salud Publica.

[11] Quezada-Pinedo HG, Ahanchi NS, Cajachagua-Torres KN, Obeso-Manrique JA, Huicho L, Grani C (2023). A comprehensive analysis of cardiovascular mortality trends in Peru from 2017 to 2022: Insights from 183,386 deaths of the national death registry. Am Heart J Plus.

[12] Navea O, Tapia V, Maluenda F, Miguel A (2023). Estratificación de riesgo del dolor torácico en el servicio de urgencia. ARS Med (Santiago).

[13] Poldervaart JM, Langedijk M, Backus BE, Dekker IMC, Six AJ, Doevendans PA (2017). Comparison of the GRACE, HEART and TIMI score to predict major adverse cardiac events in chest pain patients at the emergency department. Int J Cardiol.

[14] Six AJ, Cullen L, Backus BE, Greenslade J, Parsonage W, Aldous S (2013). The HEART score for the assessment of patients with chest pain in the emergency department a multinational validation study. Crit Pathw Cardiol.

[15] Nilsson T, Johannesson E, Lundager J, Mokhtari A, Ekelund U (2021). Diagnostic accuracy of the HEART Pathway and EDACS-ADP when combined with a 0-hour/1-hour hs-cTnT protocol for assessment of acute chest pain patients. Emerg Med J.

[16] Chacón-Diaz M, Salinas J, Doig R (2018). Estratificación del dolor torácico con el score HEART modificado y su relación con eventos adversos cardiovasculares a corto plazo. Arch Cardiol Mex.

[17] Mahler SA, Stopyra JP, Apple FS, Riley RF, Russell GB, Hiestand BC (2017). Use of the HEART Pathway with high sensitivity cardiac troponins A secondary analysis. Clin Biochem.

[18] Pérez M, Satústegui PJ, Benito E, Solans A, Fernández MT (2023). Comparación de las escalas Heart, Grace Score y los parámetros clínicos como predictores de eventos cardiovasculares en pacientes con dolor torácico en Urgencias. Rev Esp Salud Publica.

[19] Than M, Flaws D, Sanders S, Doust J, Glasziou P, Kline J (2014). Development and validation of the Emergency Department Assessment of Chest pain Score and 2 h accelerated diagnostic protocol. Emerg Med Australas.

[20] Stopyra J, Snavely AC, Hiestand B, Wells BJ, Lenoir KM, Herrington D (2020). Comparison of accelerated diagnostic pathways for acute chest pain risk stratification. Heart.

[21] Fernando SM, Tran A, Cheng W, Rochwerg B, Taljaard M, Thiruganasambandamoorthy V (2019). Prognostic Accuracy of the HEART Score for Prediction of Major Adverse Cardiac Events in Patients Presenting With Chest Pain A Systematic Review and Meta-analysis. Acad Emerg Med.

